# Assessment of mortality and marital status of schizophrenic patients over a period of 13 years

**DOI:** 10.4103/0019-5545.31595

**Published:** 2006

**Authors:** R. Ponnudurai, J. Jayakar, B.W.C. Sathiya Sekaran

**Affiliations:** * Professor and Head Department of Psychiatry, Sri Ramachandra Medical College and Research Institute (Deemed University), Chennai 600116; Former Dean, Tamil Nadu Medical Service; Director, Institute of Mental Health; Professor and Head, Department of Psychiatry, Madras Medical College, Chennai; ** Former Additional Professor Psychiatry Madras Medical College and Institute of Mental Health, Chennai; *** Professer and Head Department of Community Medicine, Sri Ramachandra Medical College and Research Institute (Deemed University), Porur 600116, Chennai

**Keywords:** Death, schizophrenia, mortality rate, marital status

## Abstract

**Background:**

Studies on standardized mortality rates of schizophrenic patients might help to increase the life span of these patients. Such data from developing countries including India are lacking. Investigations that provide clues regarding the quality of their family lives could also be beneficial.

**Aim:**

Besides mortality and causes of death, this study was also aimed to examine the marital status of a group of schizophrenic patients over a period of 13 years.

**Methods:**

Out of 121 schizophrenic patients recruited earlier for a different purpose, 60 were re-assessed after a period of 13 years with regard to their mortality and causes of death. The proportion of those who had to remain single because the onset of their illness was before they were 25 years of age were compared with those who had a later onset.

**Results:**

Seven deaths were recorded and the standardized mortality ratio (SMR) for all the age groups was 54.2. One patient who had absconded was not counted as a case of death. Furthermore, this trend of a high SMR persisted despite merging both the cohort and un-reassessed group (SMR 25.1) with and without known mortality. Among the deaths, the unnatural causes of death were noteworthy (28.57%)—1 accidental and another suicidal. Some deaths were probably due to poor general medical care. The proportion of patients who had to remain single because their onset of illness was before 25 years of age was significantly more than those with a later onset (p<0.001).

**Conclusion:**

It is speculated that strengthening the general healthcare delivery system for the mentally ill and sensitizing caregivers about the possible risky behaviours of patients might reduce the mortality.

## INTRODUCTION

Patients with schizophrenia have consistently been found to have a higher rate of natural and unnatural mortality.^1^ Mortality studies of cohorts with schizophrenia are plagued with a number of methodological problems that include insufficient sample size, failure to establish the correct cause of death, failure to follow up the entire study cohorts, reliance solely on data from case registries, etc. Furthermore, the available data from the mortality studies conducted so far might not reflect the actual position prevailing in some of the developing countries. In fact, there is a dearth of reports on the standardized mortality ratio (SMR) from such countries including India. Inadequate information in the census records, absence of national registers for schizophrenia, frequent migration of people and poor compliance of patients are some of the reasons for the lack of such data. We describe the mortality among a cohort of 60 patients with schizophrenia living in the state of Tamil Nadu, India, who were revisited by these authors after 13 years.

Since schizophrenia is known to undermine the prospects of marriage,^2^–^5^ this study also examined the marital status of this cohort.

## METHODS

One hundred twenty-one cases of schizophrenia who reported to the psychiatric outpatient department of the Government General Hospital, Chennai, from 1981 to 1982 formed the subjects of this study. After obtaining a detailed history from a close relative of the patient, the mental status examination was independently done for each of these patients by two psychiatrists, one of whom was the first author and the other a senior consultant psychiatrist. Only those who fulfilled the Feighner criteria for schizophrenia^6^ after a diagnostic consensus were selected. These subjects were actually recruited for a different analysis that has been reported elsewhere.^7^

Thirteen years after their registration, an attempt was made in 1995 to contact all the subjects to find out whether they were alive or dead, and to assess their marital status. Establishing contact with 18 subjects was easy as they were attending the outpatient services. An attempt was made to contact the remaining patients through letters written to the addresses noted in the case records and, consequently, information from the subjects and their relatives who turned up in person were obtained. In cases where letters were returned undelivered, or where nobody from the family responded after the receipt of two letters, home visits were made by an assistant to locate the patient. If the patient was located but could not be brought to the outpatient services, or the patient was reported to have died, home visits were made by the authors to obtain accurate information. However, out of the original 121 cases, only 60 could be contacted again, as the rest could neither be traced nor could any information regarding them be obtained. Hence, only these 60 subjects were considered as the cohort for this study. Besides reviewing the case records, at least one key informant who was personally acquainted with the patient (either a friend or a blood relative) was interviewed by two of the authors (RP and JJ) to supplement and confirm the information. The retrospective analysis of the case histories and symptomatology of the patients done by the above authors showed that they met the DSM-IV^8^ criteria for schizophrenia.

Evaluation of the cause of death was done by the authors by going through the case notes of the hospital where the patients were treated for their medical problems. The validity of the diagnosis made by the treating physicians and the cause of death were checked with the results of the investigations done in the hospitals. Also, the information collected from relatives pertaining to the medical illness that led to the death and the case records of the hospitals were cross-verified to arrive at the cause of death. In cases of suicide and accident, the information provided by the relatives was also cross-verified with those available with the police. After obtaining the appropriate mortality rate in the population from the census department records,^9^ the number of deaths observed were divided by the number of deaths expected to derive the standardized mortality ratio (SMR); 95% confidence intervals were also calculated.

The marital status of the subjects was examined after excluding those with incomplete data. The group that remained single (unmarried, divorced and separated) was compared with the married group. Chi-square test was used as the statistical test of significance. For this type of analysis, the subjects were divided into two groups, based on whether the onset of illness was before or after 25 years of age. The mean age of the cohort at the time of their first entry to the treatment facility was also evaluated.

## RESULTS

Of the 60 subjects (Males 28, Females 7), 7 died during the 13-year period of this study. Yet another subject whose whereabouts were not known for more than 10 years and hence could have been counted as a case of death as per the law of our land, was however not regarded as dead for the purpose of this study. The mean age of males at the time of first entry for management was 27 years (SD 8.7) and for females 26.7 years (SD 8.6).

### Causes of death

The causes of death for all the subjects are given in Table 1. The death of 1 male patient was uncertain, as he absconded at the age of 45 years, 10 years after entry into the treatment programme, and his fate could not be ascertained at the time of conclusion of this study. Two of the 7 deaths (28.57%) were due to unnatural causes; both were men. Of these, 1 committed suicide by jumping from the second floor, and the other accidentally slipped while drawing water into a well that did not have a protective side wall. The suicide was committed at the age of 29 years within a period of 6 years from the time of onset of the illness. Of the natural causes of death, anaemia with cardiac failure and pulmonary tuberculosis were probably chronic medical diseases.

**Table 1:** Causes of death

Cause of death	*n* (7)
Suicide	1
Accidental	1
Diarrhoea	2
Anaemia with cardiac failure	1
Pulmonary tuberculosis	1
Pyrexia of unknown origin	1

### Mortality rate

The SMR for all the age groups (54.2) was much above the average for the general population of the urban areas of Tamil Nadu, of which Chennai city is a part Table 2. Would the SMR have been lower if the high attrition rate of our sample was reduced by means of follow up at more frequent intervals? Speculation of this kind was countered by merging the cohort for whom the mortality was known with the un-reassessed group of the original sample whose mortality was unknown and hence assumed to be alive. Despite such an analysis the SMR was still high (SMR 25.1, Table 3).

**Table 2:** Mortality rate for all the age groups of the cohort

Age at entry for psychiatric treatment (years)	*n* (60)	Population death rate per 1000	Observed deaths	Expected death rate
15-20	13	1.4	1	0.0182
21-25	17	1.7	1	0.0289
26-30	12	1.9	1	0.0228
31-35	11	2.4	2	0.0264
36-40	4	2.4	0	0.0096
41-45	2	4.1	2	0.0082
56-60	1	15.0	0	0.0150

SMR: 54.2 95% CI: 14-94.4

**Table 3:** Mortality rate for the entire original sample (inclusive of those who could not be re-assessed and were assumed to be alive)

Age at entry for treatment (years)	*n* (121)	Population death rate per 1000	Observed deaths	Expected death rate
15-20	26	1.4	1	0.0364
21-25	31	1.7	1	0.0527
26-30	26	1.9	1	0.0497
31-35	19	2.4	2	0.0456
36-40	10	2.4	0	0.0240
41-45	5	4.1	2	0.0205
46-50	1	5.4	0	0.0054
51-55	0	8.0	0	-
56-60	3	15.0	0	0.0450

SMR: 25.1 95% CI: 6.5-43.7

### Marital status

A significantly larger number of subjects had to remain single when their onset of illness was before 25 years of age in comparison with those in whom the onset was later Table 4. The singles group included those who were unmarried, as well as those divorced and separated due to the illness. Nevertheless, a comparison of divorced and separated subjects with those living together did not demonstrate a statistically significant difference, despite a higher proportion of females being divorced or separated from their spouses because of mental illness.

**Table 4:** Marital status

Time of onset of illness	Remained single	Remained married
Before 25 years (*n*=31)	21	10
After 26 years (*n*=19)	2	17

*X*^2^ = 13.3067, p<0.001

## DISCUSSION

Extensive reviews of the literature^10^,^11^ have shown increased mortality in schizophrenia. The high SMR in our sample could be explained by the ignorance of and illiteracy among our patients, which dissuade them from seeking treatment at an early stage. Furthermore, as the treatment at our facility is free of cost for poor patients, our centre attracts mostly those sections of society which are vulnerable to predisposing risk factors such as ignorance, poor diet, poor hygiene, poor living conditions, inaccessibility to medical care and related issues. These factors may partly explain the deaths due to diarrhoea, anaemia with cardiac failure and pulmonary tuberculosis.

The unnatural deaths in our sample, as in other series, were due to suicide^12^ and accident.^13^–^15^ The case notes of the subject who committed suicide did not reveal the presence of suicidal ideation or history of any suicidal attempt. However, he was on irregular treatment and was not in contact with the psychiatric services for 6 months prior to the fatal act. Surprisingly, in a 16-year follow-up study reported elsewhere^16^ there was not a single case of suicide. The observation of an accidental death in our study should deter employers and caregivers from engaging patients in risky jobs. However, it is unrealistic to expect that all suicides and accidents can be prevented in those with schizophrenia, as some occur without warning or among patients who reject psychiatric advice.

We did not have sufficient information on one of our patients regarding the probable cause for his absconding. Perhaps possession of an identity card by potential absconders might help to trace them back with ease in such eventualities. Also, an organized network of community mental health agencies might be helpful in tracing such patients. However, such networks are yet to be developed in our country.

Analysis of the marital status by age of onset showed that significantly more subjects had to remain single when the age of onset of illness was earlier than 25 years. The percentage of schizophrenia patients who get married has been shown to be much lower than normal individuals or those with other psychiatric disorders by other researchers as well.^2^–^5^ These authors attributed the low marital rates to poor pre-morbid adjustment impairing the development of heterosexual relationships, social and occupational disability arising due to the illness, besides the clinical symptoms and early age of onset as observed by us. An association between mental illness and marital problems has been documented by Indian studies too.^17^ Another study from India^18^ reported that 70% of their first episode patients were married and 80% of the marriages were intact on follow up for up to 10 years.

## CONCLUSION

The findings of this study regarding unnatural causes of death and illnesses leading to death among patients with schizo-phrenia could perhaps be due to poor general medical care. Due to the authors' taking up assignments elsewhere for a few years during the study period there was a lack of follow up of the original sample at short intervals. This made it impossible to trace the frequently migrating group of patients. Inevitably, this resulted in a dropout rate of 50% of the original sample for our analysis, which is a limitation of this study. Hence, it is difficult to draw unequivocal conclusions, but it is still probable that our study strengthens the notion that schizophrenia contributes to excess mortality, since analysis after the merger of the cohort with the un-reassessed group of the original sample also showed a high SMR. At any rate, our study could serve as a pointer for more research in this area from developing countries where the healthcare delivery systems might need strengthening.
